# Bilateral horizontal gaze palsy in an 8‐year‐old girl: A rare case with *NDUFS4* gene mutation

**DOI:** 10.1002/ccr3.4748

**Published:** 2021-08-30

**Authors:** Mohammad Vafaee‐Shahi, Saeide Ghasemi, Mehran Beiraghi Toosi, Mahmoud Reza Ashrafi, Reza Shervin Badv, Ali Reza Tavasoli, Leila Tahernia

**Affiliations:** ^1^ Pediatric Neurology Department Pediatric Growth and Development Research Center Iran University of Medical Sciences Tehran Iran; ^2^ Rasool Akram Hospital Iran University of Medical Sciences Tehran Iran; ^3^ Pediatric Neurology Department Pediatric Ward Faculty of Medicine Mashhad University of Medical Sciences Mashhad Iran; ^4^ Myelin Disorders Clinic, Pediatric Neurology Division, Children's Medical Center, Pediatrics Center of Excellence Tehran University of Medical Sciences Tehran Iran; ^5^ Pediatric Intensive care department, Children's Medical Center, Pediatrics Center of Excellence Tehran University of Medical Sciences Tehran Iran

**Keywords:** gaze palsy, lactate, Leigh syndrome, mitochondria, *NDUFS4* gene

## Abstract

We report a patient with complex clinical presentation including multiple neurological symptoms and eye involvement. Upon genetic investigation, the patient was found to carry a novel homozygous mutation in the *NDUFS4* gene, thus adding to the heterogeneity of Leigh syndrome clinical presentation.

## Graphical abstract

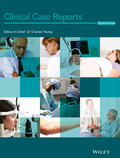

## 1 BACKGROUND

Leigh syndrome (LS) is a rare and inherited disease that is associated with progressive neurological disorders. Here, an 8‐year‐old girl is reported with bilateral horizontal gaze palsy, ataxia, and drowsiness. The laboratory test result including biochemical, hematological, immunological, infectious, and inflammatory markers and blood and cerebrospinal fluid (CSF) lactate were reported within normal values. Brain magnetic resonance imaging (MRI) revealed dorsal midbrain, bilateral putamen nuclei, and cerebellar dentate nucleus involvement. Ocular examination showed retinal atrophy and pale disk on both sides. Magnetic resonance spectroscopy (MRS) revealed an elevated lactate peak in involved areas, which suggested a mitochondrial disease. Finally, the molecular genetic test reported a homozygous *NDUFS4* gene mutation, which confirmed the presence of Leigh syndrome. Our findings indicated that various symptoms and clinical features can be found in Leigh syndrome, which could be probably due to different mutations in mitochondrial genes. Therefore, appropriate clinical and laboratory settings along with brain MRI, MRS, and genetic test analysis would be necessary for the early diagnosis.

Leigh syndrome is a rare and inherited heterogeneous disease, which is associated with progressive neurodegenerative brain damage. Diarrhea, vomiting, and dysphagia are usually the initial signs of LS in infants. Clinically, it may be also associated with multiple features, including brainstem symptoms, ataxia, hypotonia, dystonia, respiratory insufficiency, pyramidal signs, lactic acidosis in blood or cerebrospinal fluid, and hypertrophic cardiomyopathy.^1^ It is usually associated with cerebral hypotonia, movement, and mental disabilities, and respiratory failure, which results in death within 2–3 years.^2^ Weakness of limbs and loss of sensation are also common in these cases.^3^ Although the basic neuropathological features in affected children are relatively similar, there is heterogeneity in clinical, genetic, and biochemistry findings. Therefore, early diagnosis of the disease is essential to increase the survival rate in affected patients.

The underlying molecular mechanism of LS pathogenesis is mainly due to the defects in mitochondrial respiratory chain enzymes.^4^ The mitochondrial complex I (NADH: ubiquinone oxidoreductase, EC 1.6.5.3) removes electrons from NADH and passes them to the electron acceptor ubiquinone.^5^ Complex I is made of 46 structural subunits, and mutations in genes coding for such subunits can cause mitochondrial complex I deficiency, which is at the basis of Leigh syndrome (LS). LS can also be linked to mutations in other genes that code for components of the aerobic energy production system such as the pyruvate dehydrogenase complex, the ATP synthase subunit‐6 (MT‐ATP), the cytochrome‐c oxidase.^4^, ^5^ More than 35 different genes have been identified in the mitochondrial respiratory chain that is associated with Leigh syndrome. Nearly 25% of Leigh disease mutations have been detected in the mitochondrial genome. Here, we reported the first case of LS from Iran with *NDUFS4* gene mutation presented with horizontal gaze palsy.

## 2 CASE PRESENTATION

An 8‐year‐old girl was admitted to Rasoul Akram hospital with bilateral horizontal gaze palsy, ataxia, and drowsiness. The gaze palsy was initiated in the right eye during the last year and then gradually affected the left eye during the previous month. She developed an unsteady gait, drowsiness, progressive ataxia, intention tremor, and seizure during the admission time. The past medical history of the patient revealed that she was the first child of the family, from a consanguineous marriage. She had also a developmental milestone delay. She had a history of seizures around 4 years ago, without current medication therapy. Our case is the first child of the family to be the result of kinship marriage.

**FIGURE 1:**
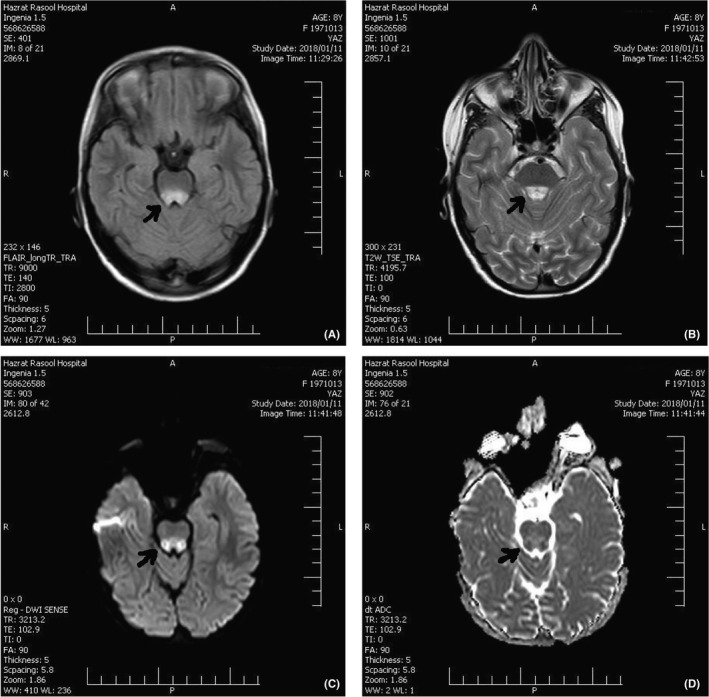
The first brain MRI. (A) Axial brain MRI (flair): Arrow shows hyperintensity in dorsal midbrain; (B) Axial brain MRI (T2): Arrow shows hyperintensity in dorsal midbrain; (C) Axial brain MRI (DWI): Arrow shows hyperintensity in dorsal midbrain; (D) Axial brain MRI (ADC map): Arrow shows restriction in dorsal midbrain involvement

The vital signs were stable during her admission time. However, she was confused but responded to verbal stimulations. Horizontal gaze palsy was detected in both eyes under ocular examinations, without any vertical gaze palsy. Nystagmus was not observed during the examination. The normal light reflection was detected in her pupils. Ocular examination revealed retinal atrophy and pale disk on both sides. Cranial nerve examination was normal. Deep tendon reflexes (DTR) were Brisk. Cerebellar examination revealed positive tandem gait, intention tremor, ataxic, and wide‐based gait. The results of finger‐to‐nose and heel‐to‐shin tests were normal. The biochemical test results, including complete blood count (CBC) and electrolytes, were reported normal. Brain magnetic resonance imaging (MRI), which was provided 1 month ago in another center, reported dorsal midbrain involvements (Figure 1). Total abdominal and pelvic ultrasounds were performed to rule out the possibility of organomegaly and malignancy. Electroencephalography (EEG) was performed to show slowing background activity prominent in the posterior area without epileptiform discharges.

On the 5th day of admission, the patient presented a generalized tonic‐clonic (GTC) seizure that lasted for 1 min. The analysis of cerebrospinal fluid (CSF) reported acceptable results for cells, biochemical tests, lactate dehydrogenase (LDH), and lactate contents. Anti‐neuromyelitis optica antibody (NMO), IgG index, and oligoclonal bands (OCB) tests were performed for the possibility of autoimmune diseases. A basic metabolic panel test was performed for the assessment of serum ammonia, lactate, pyruvate, and amino acid content with the high‐performance liquid chromatography (HPLC) method. The possibility of specific metabolic syndromes was ruled out when the metabolic results were normal. The patient received biotin, vitamin B1, B_12_, and vitamin E to evaluate the possibility of Wernicke‐Korsakoff syndrome. However, no change in signs and symptoms was observed following these treatments. Serum immunoglobulins were evaluated to rule out the possibility of ataxia telangiectasia and other immune deficiencies. The laboratory test results including ANA; anti‐ds‐DNA; serum complements (C3, C4, and C5), SSA‐Ro, SSA‐LA; RPR; AFP; anti‐GM Ab (IgM and IgA); β2 glycoproteins; anti‐phospholipids; anticardiolipin (IgM and IgG); HLA (B5 and B51); and serum ACE were within normal values. Therefore, the possibility of neuro‐Behcet disease, lupus erythematous, neuro‐brucellosis, and neuro‐sarcoidosis was declined by detecting normal laboratory results. The infectious diseases biomarkers were performed to rule out the possibility of HIV Ab, VDRL, anti‐toxoplasma Ab (IgM and IgG), wright, and 2ME. Wilson's disease was evaluated by a 24‐h urine copper test. Serum levels of vitamin B_12_ and folic acid were normal. Listeria infection was a recommended diagnosis due to midbrain involvement. So, ampicillin and gentamycin were then started empirically.

**FIGURE 2:**
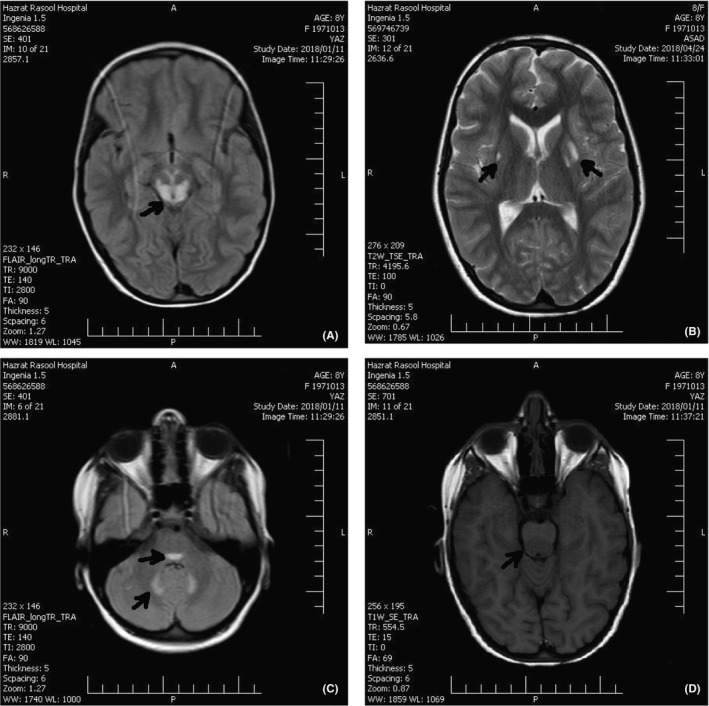
The second brain MRI. (A) Axial brain MRI (flair): Arrow shows hyperintensity in dorsal midbrain; (B) Axial brain MRI (T2): Arrows show bilateral hyperintensity in putamen nucleus; (C) Axial brain MRI (flair): Arrows show hyperintensity in dorsal midbrain and dentate nucleus of the cerebellum; (D) Axial brain MRI (T1): Arrow shows hypointensity in dorsal midbrain area

On the 10th day of admission, the second brain MRI was performed with and without contrast for the second time (Figure 2). Abnormal high signal flair and T2 lesions were detected in the medulla, midbrain, bilateral putamen nuclei, and cerebellar dentate nucleus. Corticosteroid pulse therapy was started to resolve involved areas, the increased signal in the midbrain, progressive ophthalmoplegia, and consequently the possibility of inflammatory lesions.

**FIGURE 3:**
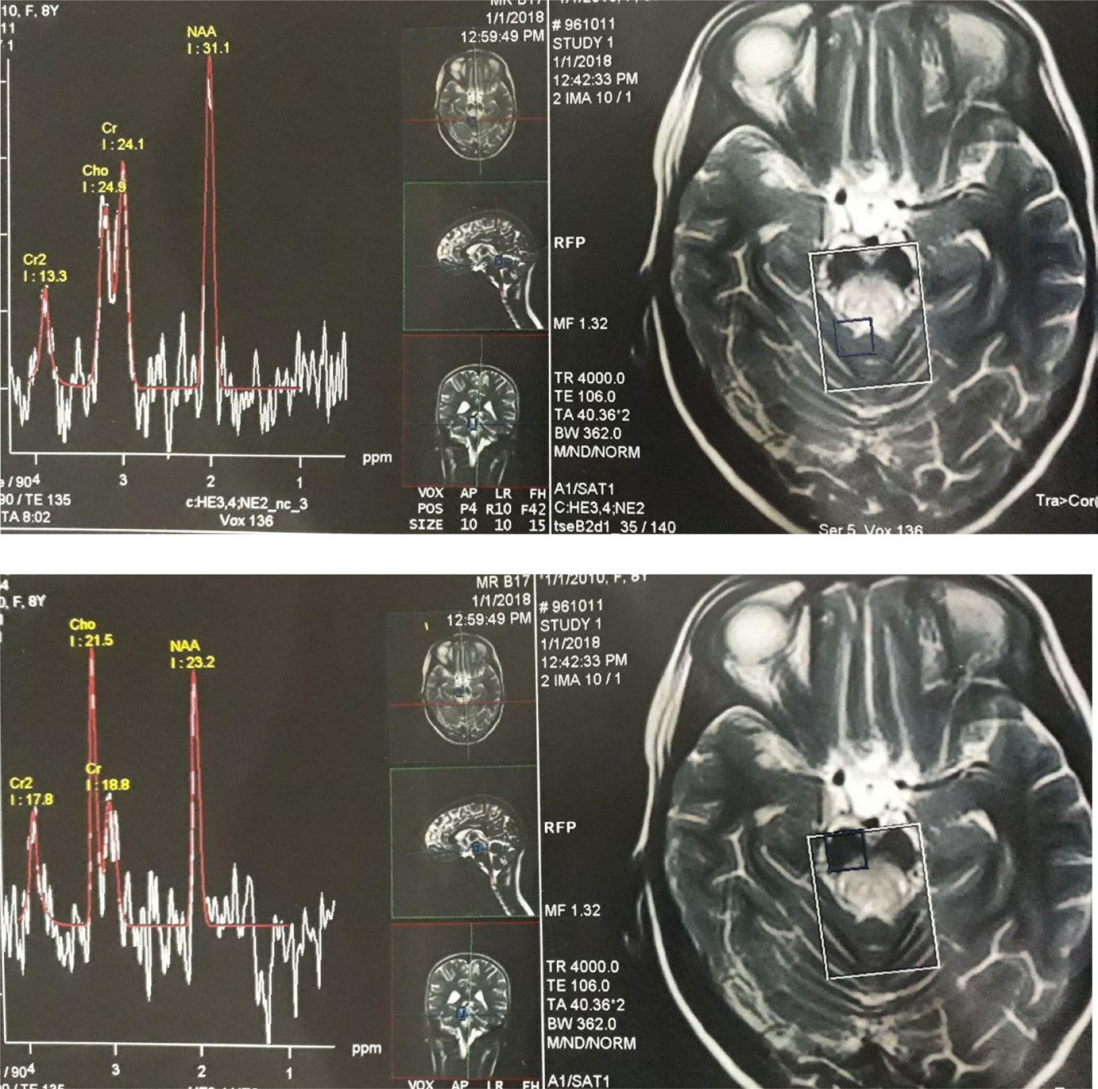
Magnetic resonance spectroscopy result. The result demonstrated an elevated lactate peak in involved areas

**FIGURE 4:**
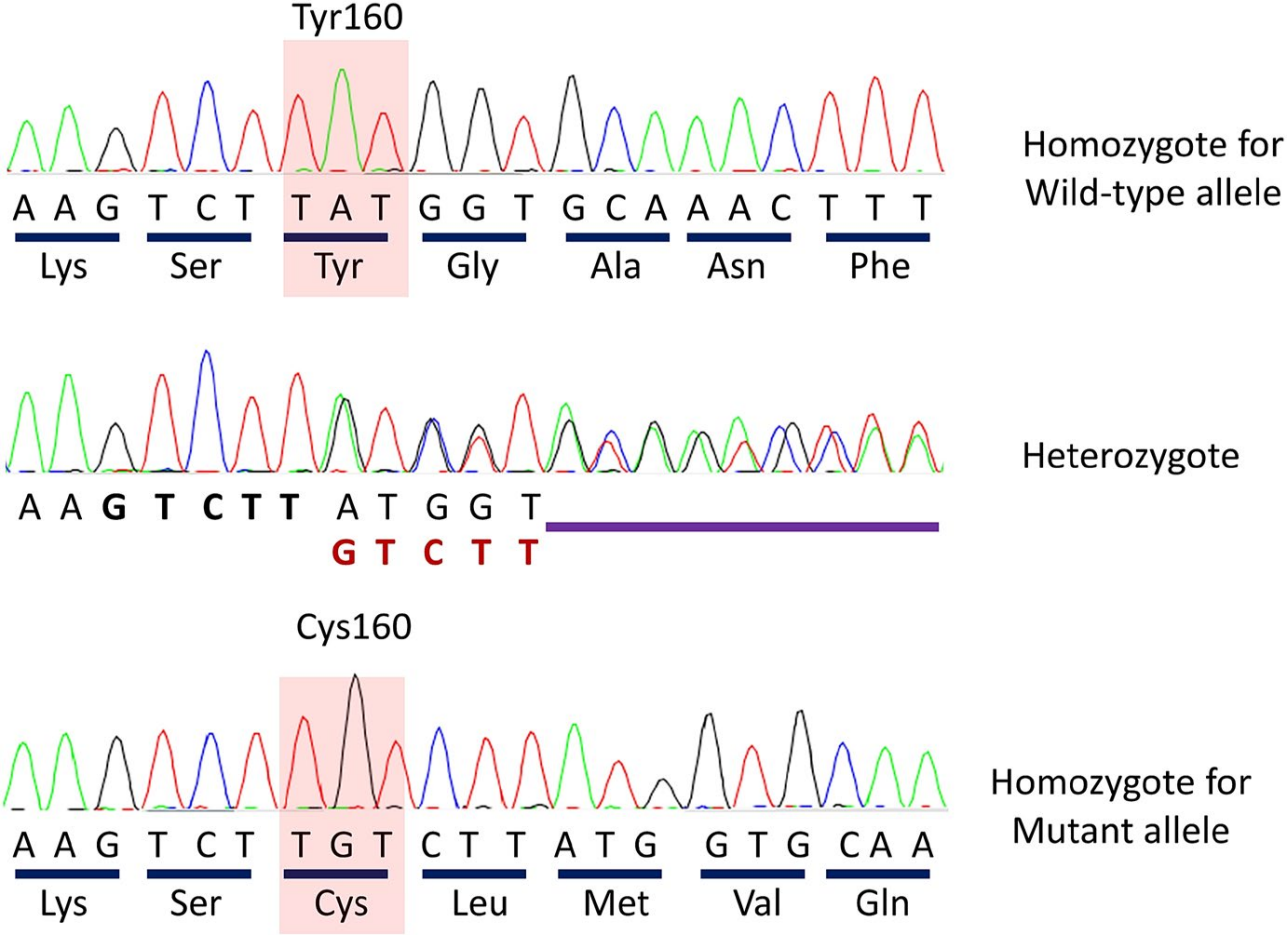
Sanger sequencing chromatograms showing the c.474‐478dupGTCTT variant in the *NDUFS4* gene. The upper sequencing trace shows the normal state in the healthy sibling, the middle trace indicates the heterozygote state in the mother, and the lower trace shows the homozygote mutant state in the affected proband of this family

On the 15th day of admission, magnetic resonance spectroscopy (MRS) demonstrated an elevated lactate peak in involved areas, which could indicate the possibility of mitochondrial disease (Figure 3). A mitochondrial treatment cocktail (including vitamin B2, vitamin B6, folic acid, L‐carnitine, and coenzyme Q) was prescribed for the patient. Gaze palsy of the eyes, ataxia, tremor, and ophthalmoplegia was improved relatively. The patient was discharged with a mitochondrial treatment cocktail and oral prednisolone (1 mg/kg/day). The patient was examined again 2 weeks after discharge. The complete improvement was observed in eye movements and gaze palsy; however, mild intention tremor and ataxia were detected during the examination time. Unfortunately, ataxia and dysarthria were gradually progressed despite the use of mitochondrial cocktail, and she lost the ability to walk after 1 year. Whole‐exome sequencing was done to evaluate any genetic disorder. In this work, the whole‐exome sequencing technique revealed a novel variant, c.474‐478dupGTCTT, in the *NDUFS4* gene. This homozygous mutation leads to a shift in the reading frame starting at codon 160 and could be responsible for the symptoms of Leigh syndrome. The new frame reading ends in a stop codon 30 downstream. Finally, the genetic study showed a homozygous mutation in the *NDUFS4* gene, confirming the diagnosis of Leigh syndrome. Sanger sequencing of her family revealed that both parents were a carrier for this mutation and were in heterozygote state (Figure 4).

## 3 DISCUSSION

Leigh syndrome is a rare progressive neurological disorder typically in childhood that can be inherited as X‐linked recessive, autosomal recessive, and mitochondrial trait. The incidence of this syndrome is estimated at approximately 2 in 100,000 births. Leigh syndrome may present early in life with multiple characteristics such as weakness, drowsiness, dystonia, psychomotor regression, cerebral hypotonia, ataxia, developmental milestones delay, and signs of brainstem involvement, seizures, visual loss, and tachypnea. Some studies reported the occurrence of sleep apnea syndrome and abdominal symptoms in these patients.^6^ However, the exact mechanism is unknown. Progressive respiratory failure is one of the main leading causes of mortality among LS cases.^7^ Elevated blood and CSF lactate, as well as pyruvate levels, can be found upon laboratory tests analysis. However, neuroimaging is a valuable tool for the diagnosis of Leigh syndrome.^8^

The present case was referred to our hospital with the chief complaint of progressive gaze palsy and also ataxia, intention tremor, and drowsiness. Her ocular examination revealed retinal atrophy and pale disk. In a recent study, Jabeen et al., ^9^ reported a 37‐year‐old female patient with Leigh syndrome who was admitted with similar ocular symptoms including bilateral horizontal gaze palsy and ataxic gait. Ocular involvement is frequently reported in patients with Leigh syndrome. Many patients with Leigh syndrome may suffer from ophthalmoplegia, a condition which is associated with weakness or paralysis of the muscles responsible for eye movement.^10^ Some studies have reported nystagmus, optic atrophy, ptosis, strabismus, and pigmentary retinopathy in patients with Leigh syndrome.^10^, ^11^ Our case had also a past medical history of delayed development and seizure. Most of the reported LS patients like the present case excluded infections, autoimmune diseases, and toxins as the causes of presented symptom. The second brain MRI reported dorsal midbrain, cerebellar dentate nucleus, and bilateral putamen nuclei involvement. These symptoms pointed toward a neurodegenerative disorder. A basic metabolic panel test result showed normal serum ammonia, pyruvate, and amino acid contents and normal blood and CSF lactate. Magnetic resonance spectroscopy revealed an elevated lactate peak in involved areas, which suggested a possible mitochondrial disease. Eventually, the molecular genetic test analysis confirmed the Leigh syndrome. She initially responded to mitochondrial treatment cocktail and significant improvement was observed in clinical symptoms. The complete improvement was observed in eye movements and gaze palsy; however, mild intention tremor and ataxia were detected during the examination time. Unfortunately, ataxia and dysarthria gradually progressed, and she lost the ability to walk after 1 year. Several studies showed similar initial responses to mitochondrial treatment cocktail (including vitamin B2, vitamin B6, folic acid, L‐carnitine, and coenzyme Q) in different cases.^9^, ^11^

Molecular genetic studies illustrated that Leigh syndrome can result from defects or mutations in mitochondrial enzymes such as pyruvate dehydrogenase (*PDHA1* gene mutations),^12^ cytochrome C oxidase complex (*SURF1* gene mutations in the nuclear genome), ATP synthase subunit‐6, and subunits of complex‐I.^13^ Recent evidence identified 24 known mutations in mitochondrial genes and 21 in nuclear genes in Leigh syndrome.^14^ Here, we found *an NDUFS4* gene mutation that was associated with Leigh syndrome. This gene is a nuclear‐encoded accessory subunit of mitochondrial complex I (NADH: ubiquinone oxidoreductase). This complex removes electrons from NADH and transfers them to the electron acceptor ubiquinone. Therefore, mutations in the *NDUFS4* gene can cause mitochondrial respiratory chain deficiencies. More recently, Ortigoza‐Escobar et al.,^15^ reported a patient with *NDUFS4* gene mutation related to Leigh syndrome.

These data have suggested that various symptoms and clinical features could be found in Leigh syndrome. Variations in the disease severity and the number of clinical features are probably due to different mutations in mitochondrial genes. Therefore, further studies are necessary to evaluate the relationship between genotype and phenotype. Since consanguineous marriage is common in Iran, there may be a relationship between the incidence of Leigh syndrome and consanguineous marriage. Therefore, further considerations are required to evaluate the correlation, and premarital genetic counseling and education may be helpful.

## 4 CONCLUSION

Our findings indicated that various symptoms and clinical features can be found in Leigh syndrome, which could be probably due to different mutations in mitochondrial genes. Therefore, appropriate clinical and laboratory tests along with brain MRI, MRS, and genetic test analysis would be necessary for the early diagnosis. Our patient was born from parents with consanguineous marriage, which highlighted the possible relationship between familial marriage and the incidence of Leigh syndrome. Since consanguineous marriage is popular in Iran, premarital genetic counseling and education may be helpful.

## CONFLICT OF INTEREST

None declared.

## AUTHOR CONTRIBUTION

VM, AM, BM, BR, and TA analyzed and interpreted the patient data regarding the neurologic disease. TL and GS performed to follow‐up the patient and were major contributors in writing the manuscript. All authors read and approved the final manuscript.

## ETHICAL APPROVAL

This case study was accredited by the Ethical Committee of Iran University of Medical Sciences.

## Data Availability

The datasets used during the current case study are in the manuscript and https://www.dropbox.com/home/NDUFS4.
